# Visceral adiposity, insulin resistance and cancer risk

**DOI:** 10.1186/1758-5996-3-12

**Published:** 2011-06-22

**Authors:** Claire L Donohoe, Suzanne L Doyle, John V Reynolds

**Affiliations:** 1 Department of Surgery, Trinity Centre for Health Sciences, Trinity College Dublin/St James' Hospital, Dublin 8, Ireland

## Abstract

**Background:**

There is a well established link between obesity and cancer. Emerging research is characterising this relationship further and delineating the specific role of excess visceral adiposity, as opposed to simple obesity, in promoting tumorigenesis. This review summarises the evidence from an epidemiological and pathophysiological perspective.

**Methods:**

Relevant medical literature was identified from searches of PubMed and references cited in appropriate articles identified. Selection of articles was based on peer review, journal and relevance.

**Results:**

Numerous epidemiological studies consistently identify increased risk of developing carcinoma in the obese. Adipose tissue, particularly viscerally located fat, is metabolically active and exerts systemic endocrine effects. Putative pathophysiological mechanisms linking obesity and carcinogenesis include the paracrine effects of adipose tissue and systemic alterations associated with obesity. Systemic changes in the obese state include chronic inflammation and alterations in adipokines and sex steroids. Insulin and the insulin-like growth factor axis influence tumorigenesis and also have a complex relationship with adiposity. There is evidence to suggest that insulin and the IGF axis play an important role in mediating obesity associated malignancy.

**Conclusions:**

There is much evidence to support a role for obesity in cancer progression, however further research is warranted to determine the specific effect of excess visceral adipose tissue on tumorigenesis. Investigation of the potential mechanisms underpinning the association, including the role of insulin and the IGF axis, will improve understanding of the obesity and cancer link and may uncover targets for intervention.

## Methodology

Relevant medical literature was identified from searches of PubMed and references cited in appropriate articles identified. Search terms used included: obesity, overweight, cancer, adipose tissue, inflammation, insulin, metabolic syndrome, adipokines and sex steroids. More detailed search terms were used following identification of relevant mechanisms and to identify epidemiological studies. All meta-analyses addressing cancer incidence with respect to body mass index were identified by PubMed searches. Meta-analyses of cohort or nested case control studies only were included. When available, meta-analyses were preferentially cited as epidemiological evidence. Selection of other articles was based on peer review, journal and relevance. Where possible, review articles from high impact factor peer-reviewed journals were cited.

## Introduction

The World Health Organisation defines obesity as an abnormal or excessive fat accumulation in adipose tissue, to the extent that health is impaired. The classification of obesity for epidemiological purposes defines overweight as body mass index (BMI) greater than 25 kg/m^2 ^and obesity as BMI greater than 30 kg/m^2 ^[1] (Table 1). The obese state is increasingly more prevalent in Western society and in some countries is the most prevalent body composition [2,3].

**Table 1:** Body Mass Index as a predictor of risk to health, WHO (2004)

Classification	BMI(kg/m^2^)
Underweight	< 18.50
Normal Weight	18.50-24.99
Overweight/Pre-Obese	25.00-29.99
Obese Class I	30.00-34.99
Obese Class II	35.00-39.99
Obese Class III	≥ 40.00

BMI, body mass index

**Figure 1:**
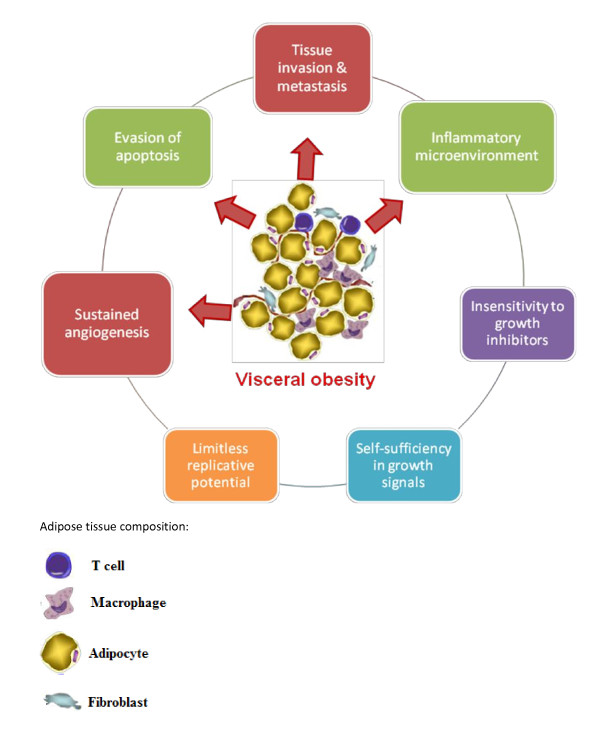
Hallmarks of cancer and potential role of visceral obesity in tumorigenesis

Adipose tissue is principally deposited in two compartments - subcutaneously and centrally (Figure 1). It is thought that centrally deposited, or visceral, fat is more metabolically active than peripheral subcutaneous fat [4-6]. Visceral adipose tissue is largely comprised of omental adipose tissue but also includes other intra-abdominal fat sources such as mesenteric fat. Visceral adipose tissue secretes a number of adipokines and cytokines leading to a proinflammatory, procoagulant and insulin resistant state collectively known as the metabolic syndrome [7]. The importance of adipose tissue location in terms of dysmetabolism risk is evident as central obesity is more strongly associated with increased risk of insulin resistance, the metabolic syndrome and cardiovascular diseases than BMI alone [8]. For any given amount of total body fat, the subgroup of individuals with excess visceral fat (versus subcutaneous fat) is at higher risk of developing insulin resistance [9] and the features of the metabolic syndrome [10]. Visceral fat remains more strongly associated with an adverse metabolic risk profile even after accounting for the contribution of other standard anthropometric indices [11]. These systemic effects exerted by visceral adiposity are putatively involved in cancer biology [12] and are the focus of much research.

### Measuring Visceral Adiposity

Commonly used BMI cut-off values to diagnose obesity have high specificity, but low sensitivity to identify adiposity, as they fail to identify those with excess body fat [13]. Waist circumference (WC) has been shown to be an accurate predictor of visceral fat, either alone or in combination with BMI [14]. WC directly reflects total abdominal fat mass [15,16] and gender and ethnic specific cut-offs can be used as reliable proxy values to predict an increased risk of the metabolic disease However, WC fails to quantify the visceral and subcutaneous fat compartments individually and there are no clearly defined cut-off values for an increase in cancer risk. Computed tomography (CT) imaging is the gold standard for the measurement of adiposity, since this allows for direct quantification of adipose tissue and can distinguish between visceral and superficial fat compartments [17]. In terms of cardiovascular risk, peripheral fat accumulation (as measured by leg subcutaneous fat deposition) may be protective [11]. Therefore, waist:hip ratio (WHR) measurements may be more relevant in determining CVD risk than WC alone. Whether this is the case for cancer risk is not yet known.

### Gender influence on visceral adiposity

Gender influences fat distribution, with women having a greater amount of peripherally located subcutaneous fat and men having a greater amount of centrally located visceral fat [8]. Men store about 20-30% of their total body fat in the visceral compartment irrespective of their obesity status [18]. Women do not accumulate significant quantities of visceral fat until a moderate level of obesity is reached and non-obese women have very small amounts of visceral fat [19]. On average, men have twice as much visceral fat as women and demonstrate a high prevalence of the obesity-related metabolic diseases and the metabolic syndrome [20,21].

The cause of this gender difference is uncertain but may be related to the higher amount of hepatic free fatty acid delivery derived from lipolysis from visceral fat that has been observed in women compared to men [22]. In addition, oestrogen may be a key regulator in mediating differences in adipose tissue distribution between men and women [8]. Pre-menopause, women have higher levels of subcutaneous adiposity and there is a lower incidence of obesity associated dysmetabolism. However, after menopause, circulating levels of oestrogen fall and adipose tissue distribution becomes more "male-like" with increased amounts of visceral adiposity and a subsequent increased risk of obesity-related metabolic disorders [8].

## Obesity and cancer: epidemiology evidence

Epidemiological studies provide convincing evidence of an association between obesity and cancer development at numerous sites: oesophagus (adenocarcinoma), pancreas, colorectum, breast (postmenopausal), endometrium and kidney [23]. The largest meta-analysis to date includes 282,000 patients from prospective observational studies with over 133 million person-years of follow-up [24]. This shows that high body mass index is associated with an increased incidence of many types of cancer. The association is modest with risk estimates of 1.1 to 1.6 per 5 kg/m^2 ^incremental increase in BMI. This 5 kg/m^2 ^increase in BMI corresponds to 15 kg weight gain in men and 13 kg in women with an average BMI of 23 kg/m^2^. The associations between obesity and cancer are sex and site specific but broadly consistent across geographic populations [24]. Emerging evidence suggests that weight loss following bariatric surgery leads to a reduction in cancer incidence [25]. The association also fulfils many of Hill's postulates [26] (plausible biological mechanisms, consistent associations, sufficient latency period, reversibility) indicating that the obesity is likely to be causal.

Furthermore, a prospective study of 900,000 adults in the United States reported that obesity could account for 14% of all deaths from cancer in men and 20% in women [27]. Those with a BMI greater than 40 kg/m^2 ^had a death rate 52% higher in men and 62% higher in women when compared to those of normal weight. This indicates that obesity may also affect outcomes following cancer diagnosis and this finding is supported by other studies which control for increased peri-operative mortality rates amongst the obese [28-31].

There appears to a sex differential with respect to risk of cancer development with men having a higher risk of developing cancer at increased BMI than women [24,32]. This may be due to the differing hormonal milieu in females or it may reflect the fact that BMI poorly reflects central adiposity in females. Since females generally only deposit central adipose tissue once total fat volumes are raised, overweight BMIs do not correspond with visceral fat volume in females as they do in males which may account for the differences in cancer risk seen when BMI is used to determine obesity status.

In studies that use measures of visceral adiposity such as WC or VFA, visceral adiposity is associated with increased risk of cancer development [32-34]; is a stronger predictor of cancer risk than BMI [32] and the cancer risk is similar in males and females [32,33]. Further, larger studies using measures of visceral adiposity across cancer sites are awaited in order to clarify whether there is a clear differential effect of visceral versus subcutaneous obesity.

Visceral adiposity (and not subcutaneous adiposity) is associated with development of features of the metabolic syndrome (taken as a proxy measure of a dysmetabolic profile in viscerally obese patients)[35]. Most of the components of the syndrome, alone [36,37] or in combination [38-40], have been individually link with cancer development at various subsites. A prospective international population-based study of 580,000 people (Me-Can Study) is underway to identify whether the metabolic syndrome is independently associated with cancer development [41]. Initial findings suggest that a combination of components of the metabolic syndrome is associated with risk of colorectal cancer development (Men: RR: 1.25 (95% CI; 1.18-1.32; Women: RR 1.14 (95% CI; 1.02-1.18)[42], endometrial cancer (RR 1.37, 1.28-1.46)[43], bladder cancer in men (RR: 1.1, 1.01-1.18)[44] and pancreatic cancer in women (RR,1.58; 1.34-1.87)[45].

## Mechanisms underlying obesity and tumorigenesis

Long thought of as inert, adipose tissue, particularly visceral fat [46], is an important metabolic tissue which secretes factors systemically that alter the immunological, metabolic and endocrine milieu and promote insulin resistance [4]. The obese state may be thought of as a pro-tumorigenic environment which can act to facilitate tumour development by promotion of the acquisition of some of the hallmarks properties that characterise cancerous lesions (Figure 1)[47,48].

### Paracrine mechanisms

**Figure 2:**
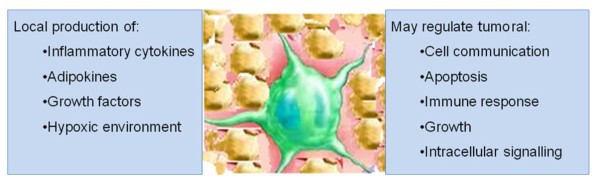
Paracrine mechanisms linking adipose tissue and cancer development

Adipose tissue may act in both a paracrine and systemic manner. At a local level, adipose tissue is involved in a number of mechanisms which may promote tumor development (Figure 2). Obese mice have reduced levels of oxygen within their epididymal adipose tissue [49]. The tumor microenvironment in solid tumors is often characterised by low oxygen tensions and hypoxia within the peri-tumoral fat may promote tumor-site hypoxia. Hypoxia upregulates the hypoxic-inducible factor (HIF-1α) which can lead to altered expression in over 60 target genes involved in angiogenesis, glycolysis, cell proliferation and apoptosis, leading to cellular adaptation to low oxygen conditions [50]. Hypoxia has been associated with metastasis and poor prognosis [51] and also induces pro-angiogeneic and inflammatory cytokine secretion [52-54].

Inflammatory cytokines produced in adipose tissue can upregulate nuclear factor-κB (NFκB) which leads to an increase in nitric oxide (NO), a substrate for reactive oxygen species (ROS). Cytokines and ROS can contribute to insulin resistance and the resultant excess circulating glucose, free fatty acids and insulin can further induce inflammation [55]. The key inflammatory pathways NFκB and STAT3 are activated by adipose tissue products leading to transcription of genes which mediate proliferation, invasion, angiogenesis, survival and metastasis [56].

The observation that epithelial tumor cell growth is enhanced by injection into fat pads rather than subcutaneously [57] supports the hypothesis that chemokine production within the adipose tissue provides conditions which enhance tumor cell growth. A proteomic study of mammary fat revealed the production of a wide variety of proteins involved in diverse processes such as cell communication, growth, immune response, apoptosis and numerous signalling molecules including hormones, cytokines and growth factors [58].

While fat which surrounds individual organs may act in a paracrine manner to influence tumor development or progression, this mechanism is less fully investigated and most research efforts have concentrated on systemic alterations in obesity and how these may influence cancer development and progression. Adipokine production and inflammatory alterations in the obese state described in detail hereafter may act to influence tumorigenesis in either a systemic or paracrine manner or a combination of both.

### Systemic mechanisms

**Figure 3:**
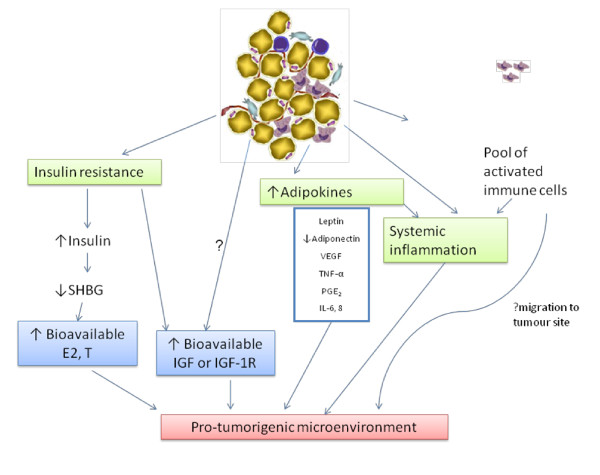
Systemic alterations in visceral adiposity which may contribute to a pro-tumorigenic microenvironment.

Systemic alterations in obesity include chronic systemic inflammation, increased adipokine production and an altered immunological status (Figure 3). Additionally, there are associated changes in the sex hormone profile. Insulin resistance develops as a consequence of visceral adiposity and there is a rise in insulin production, which may be associated with activation of the insulin-like growth factor (IGF) system. All of these changes which occur in tandem with the development of obesity have the ability to interact with each other. It is this altered systemic milieu which is thought to fuel cancer development and progression.

#### Chronic inflammation and adipokines

Excess adipose tissue results in elevated levels of pro-inflammatory adipokines, resulting in an imbalance between increased inflammatory stimuli and decreased anti-inflammatory mechanism leading to persistent low-grade inflammation [46,59,60]. The level of adipokine production from adipose tissue is strongly influenced by immune cell populations present in adipose tissue [4,61-63]. Adipose tissue in obese people is infiltrated with macrophages and the number of macrophages correlates with the degree of adiposity [64]. Peripheral monocytes are recruited by monocyte chemoattractant protein (MCP)-1 and TNF-α, and can differentiate into activated macrophages [65]. Pre-adipocytes also have the ability to differentiate into macrophages [61]. The products of activated macrophages can impact on adipocyte function and are postulated to be involved in altering adipose tissue glucose handling and thus contribute to insulin resistance [66,67]. Research has shown that co-culture of adipocytes with macrophage-conditioned media causes increased adipokine and inflammatory cytokine production by adipocytes [68], further supporting this hypothesis.

Insulin can modulate adipokine production and interacts with two of the most abundant adipokines: leptin and adiponectin. Insulin is a positive regulator of leptin and increases its gene expression to suppress appetite [69]. Adiponectin acts as an insulin sensitising agent [69]. In addition to modulation of insulin sensitivity, these adipokines can directly affect tumor cells [70]. Adiponectin is anti-tumor: it increases apoptosis[71], inhibits proliferation, inflammation and angiogenesis [72] and can prevent the interaction of growth factors with their receptors [73]. There is a consistent inverse relationship with cancer incidence and circulating adiponectin [74]. The pro-tumor effects of leptin are the direct opposite of those of adiponectin [74], although the epidemiological association between circulating levels and cancer risk is less consistent [75]. Circulating levels of leptin positively correlate and adiponectin levels negatively correlate with all measures of obesity (BMI, WC and visceral fat area)[76-78].

Thus altered adipokine production by adipose tissue, and in particular inflammed visceral adipose tissue, may influence the tumor microenvironment. Obese rat models have increased inflammatory transcription factor expression (TNFα and NFκB) in their tumors [79]. Furthermore, there is emerging evidence that adipose stromal cells may be a source of stromal cells in tumor microenvironments. Early adipocyte precursor cells can differentiate into stromal cells [80]. In animal models of obesity, adipose stromal cells and adipose endothelial cells from inflamed visceral adipose tissue migrate to tumor sites [81]. Stromal cells in the tumor microenvironment promote angiogenesis and support tumor progression [82]. There is also evidence that visceral adiposity can influence a patient's treatment outcome, with a study demonstrating increased visceral fat area to be an independent predictor of outcome after first-line bevacizumab treatment in colorectal cancer [83]. This finding indicates that angiogenic factors produced by visceral fat may influence tumor progression and response to chemotherapy and is mirrored in animal models as adiponectin, which is reduced in visceral obesity, inhibits tumor growth by reduced neovascularisation[72]. The mechanism(s) for this resistance may uncover important information on how obesity influences the tumor microenvironment.

#### Sex hormones

Epidemiological studies have suggested a difference in the influence of obesity on cancer development between men and women [27]. While some studies are inconsistent, the risk of colorectal cancer in post-menopausal women does not seem to be related or only weakly associated with obesity [84,85]. It has been hypothesised that different distributions of adipose tissue between men and women accounts for the difference [86]. Whether this is mediated by affecting circulating sex hormone levels is not fully understood. Adiposity is inversely related to testosterone concentration in men but positively related in women [87]. Excess adipose tissue leads to increased conversion of androgenic precursors to oestradiol by increased aromatase activity [88]. In endometrial cancer, oestradiol increases cell proliferation via inducing a local increase in IGF-1 [89].

Different influences of circulating oestrogen levels may influence cancer development [90]. Chronic hyperinsulinaemia may promote tumorigenesis in oestrogen-sensitive tissues as it reduces circulating sex-hormone binding globulin and thus increases bioavailable oestrogen [27,91-93]. The association between obesity and postmenopausal breast cancer risk is accounted for by the increased serum oestradiol levels as obesity increases [94] and since in females, most fat deposition occurs peripherally, this fat area may be of more relevance to endometrial and post-menopausal breast cancer risk.

#### Insulin resistance

Levels of adipose tissue affect the body's handling of glucose [95]. Adipokines are thought to be involved in the pathogenesis of insulin resistance [96,97] and as previously mentioned insulin can modulate adipokine activity. High concentration of cytokines produced by adipose tissue, such as TNF- α, IL-6, IL-1β, and low concentrations of adiponectin, have deleterious effects on glucose homeostasis leading to chronic hyperinsulinaemia and insulin resistance in Type 2 Diabetes Mellitus [98,99].

Insulin resistance is an adaptive response to raised circulating free fatty acids (FFAs) and thus is related to the extent of visceral adipose tissue deposits [100]. Raised FFAs shift metabolism of liver, muscle and other tissues towards lipid deposition and oxidation and away from gluconeogenesis and glycolysis. Insulin secretion rises to compensate for the decreased capacity to handle glucose. Despite this there is a decreased expression of insulin-receptor levels and reduced intracellular insulin signalling in response to insulin receptor binding [101]. Subcutaneous adipose tissue takes up free fatty acids and stores the excess calories more readily than visceral adipose tissue [102]. Rates of lipolysis are higher in visceral adipocytes than superificial [11], due to the anti-lipolytic effect of insulin being increased in subcutaneous adipose tissue [46]. Therefore, the risk of developing insulin resistance is related to the size of visceral fat deposits.

Several epidemiological studies have shown that insulin resistance status, characterised by hyperinsulinaemia, is associated with an increased risk for a number of malignancies, including carcinomas of the breast, prostate and colon[103-106]. Per 1 mmol/l increment in glucose from data of the MeCan study cohort has revealed an increase risk of incident cancer in men (RR 1.05, 1.01-1.1) and women (1.11, 1.05-1.16) and a further increased RR for fatal cancer (Men: 1.15, 1.07-1.22; Women: 1.21,1.11-1.33)[103].

## Insulin and cancer

Insulin can act as a mitogen and has been associated with several cancers[107]. The tumorigenic effects of insulin could be directly mediated by insulin receptors in the pre-neoplastic target cells, or might be due to related changes in endogenous hormone metabolism, secondary to hyperinsulinaemia [100]. Epidemiological studies are hampered by the heterogeneity of diabetic patients with respect to their degree of glycaemic control which will influence their circulating insulin levels and hence making correlations with cancer development difficult.

Epidemiological studies have shown that serum C-peptide, as a proxy measure of insulin release, is associated with increased risk of cancer of the colorectum, post-menopausal breast, pancreas and endometrium [108] and that type 2 diabetes is associated, independent of obesity, with breast, pancreas, kidney, endometrial, colorectal and bladder cancer [109]. Cohort studies have demonstrated increase risk of colorectal cancer in those with insulin resistance,[38,40,110] the metabolic syndrome [111] and type 2 diabetics [112]. The risk of cancer-related mortality is increased in those with high insulin levels or insulin resistance and cancers of the breast [113], prostate [114] and colorectum[115]. Colorectal cancer incidence is higher in Type 2 diabetics treated with insulin [116].

*In vitro *studies support epidemiological data in that insulin increases the neoplastic proliferation of cell lines at both physiological and pharmacological doses [117] and the insulin receptor is commonly expressed in human neoplasms. Under investigation at present is whether there are differential effects downstream signalling effects in normal or transformed epithelial cells compared to insulin-responsive tissues (such as fat, liver and muscle) with receptor activation resulting in cell survival and proliferation rather than altered energy metabolism [118]

## Insulin-like growth factors, obesity and cancer

The interaction between insulin, body fat and the IGF axis is less well understood. It has been proposed that the IGF system mediates the effect of hyperinsulinaemia and is more relevant to cancer development and progression than insulin (Figure 2). The insulin-like growth factors are involved in enhancement of cell proliferation, differentiation and apoptosis and have been implicated in tumorigenesis [119,120]. Levels of IGF are influenced by circulating insulin levels which alter the level of IGF binding protein 1 and 2 (IGFBP1 & 2) increasing bioavailability of IGF [121].Waist circumference and waist:hip ratio are inversely associated with IGFBP1 levels in healthy females[122]. The collective findings from a number of studies show that IGFBP1 & 2 is inversely associated with body fat and insulin levels and that there does not appear to be a direct correlation between total IGF1 levels and body fat or insulin [123]. Rather, there is a non-linear relationship between BMI and total IGF-1 levels (with the highest levels in those with a BMI up to 27 kg/m^2^[124]) However, the relationship between measures of visceral adiposity and IGF-1 levels is unclear. In addition, there is a debate as what constitutes bioavailable IGF-1 (total or free fractions) and whether IGFBPs and their relationship to total IGF-1 levels should be taken into account.

### The IGF axis

The IGF axis is a multifunctional system with a variety of molecular and biological effects. To date, 15 molecular functions and 29 biological processes have been linked to the IGF-1R in the gene ontology database (http://www.geneontology.org). Proteins from the axis are ubiquitously expressed but at different levels in different tissues and with varying roles in each tissue. The IGF axis can have auto-, para- and endocrine effects. The main biological processes that the IGF axis is involved with can be summarised as: control of normal growth [118,125] (and perhaps lifespan [126]); maintenance of tissue homeostasis [127] and a differentiated phenotype [128]; alteration in the balance of proliferation and apoptosis [129]; angiogenesis, cell adhesion, migration and wound healing [129].

IGF-1 and IGF-2 are bound by 6 high affinity binding proteins (IGFBP1-6) and other low affinity binding proteins (IGFBP-related proteins). IGFBP1-5 have higher affinities for IGF-1, whereas IGFBP6 has a higher affinity for IGF-2 [130]. IGFBPs stabilise and prolong the half-life of IGFs and by binding IGFs prevent their binding to receptors. IGFs are released from IGFBPs by dissociation or protease-mediated IGFBP cleavage [130]. Thus IGFBPs alter the bioactivity of IGF and in some circumstances act to increase the bioactivity of IGF. This is thought to occur by IGFBPs binding IGFs in proximity to their receptor and acting to concentrate IGFs at receptors and through the slow release of the growth factors can influence the duration of signalling via the receptor [130]. Another hypothesis is that IGFBP2 binds an integrin-linked kinase to increase IGF bioactivity [118]. The tumor suppressor p53, vitamin D, anti-oestrogens, retinoids and TGFβ reduce the bioactivity of IGFs by increasing secretion of IGFBPs [130]. IGFBPs may also have independent effects on proliferation, adhesion and motility. Certain IGFBPs modulate Wnt signalling which is involved in differentiation and this modulation is influenced by local concentrations of IGF ligands [131]. Thus the IGF system is highly regulated via a dynamic system of binding proteins which influence growth factor stability, receptor binding and duration of receptor activation.

IGF-1R is a transmembrane heterotetrameric protein encoded by the IGF-1R gene located on chromosome 15q25-q26. IGF-1R is composed of two α and two β subunits. Due to structural homology with the insulin receptor it can heterodimerize with it. The IGF-1R receptor binds (ranking from high to low affinity): IGF-1, IGF-2 and insulin[132]. IGF-1R is a tyrosine kinase receptor which initiates intracellular signalling upon receptor activation by autophosphorylation and stimulation of tyrosine kinase activity, leading to recruitment and phosphorylation of the insulin-receptor substrate-1 (IRS-1). These receptor substrates activate two main signalling pathways: PI3K-AKT and RAS-Raf-MAPK which have multiple effects on gene regulation and protein expression, activation and translocation [129]. The availability, location and ratios of receptor substrates influence cellular responses to receptor activation and may also alter the balance of IGF signalling and insulin signalling when heterodimers of IGF/insulinR are bound. Several mechanisms of crosstalk which influence IGF-1R receptor function have been describes including heterodimers with EGFR[133] and SOCS interactions influencing the Jak-Stat pathway[134].

### IGF axis and cancer

Two of the hallmarks of cancer [47] are limitless replication and evasion from apoptosis (Figure 1). The IGF axis is a central regulator of growth and survival. It has been found that IGF-1R plays a role in the establishment and maintenance of cellular transformation [135]. IGF-1R or its ligands are often over-expressed in human tumors [136,137] and its action protects against apoptosis and favours invasion and metastasis [119,120,129]. Activation of IGF-1R can promote cell migration and the redistribution of E-cadherin and α- and β- catenins from adherens junctions into the cytoplasm [138]. IGF-1 also modulates the activities of integrin-coupled proteins (FAK, p130, Cas and paxillin) through dephosphorylation [139].

The IGF-1R is commonly expressed by neoplastic cell lines and human cancers and on circulating tumor cells [136,137,140]. However, gene amplification is not commonly associated with protein overexpression or ligand-independent activation [118]. The IGF-1R has been found to be essential for oncogenic transformation in some cellular systems. Mouse fibroblasts cannot be transformed by the oncogenes: SV40 T antigen, papillomavirus E5 and Ras overexpression if they lack the IGF-1R [135]. Stable (constitutive) activation of IGF-1R is insufficient to cause mammary epithelial cell transformation in mouse models [141].

Many cell lines are mitogenically responsive to physiological concentrations of IGFs [132]. Increased proliferation in response to raised IGF levels may fuel the development of early cancers. Using prostate cancer as a model, there appears to be an increased likelihood of progression to clinically detectable malignancy in patients with higher IGF-1 levels such that baseline IGF-1 level predicts progression to prostate cancer more accurately than prostate-specific antigen in screened populations [142,143]. *In vivo *animal models using natural occurring mutations associated with low IGF levels [144,145] or genetic manipulations to influence ligand levels [146,147], result in variability of neoplastic growth related to IGF activity. Animal models have shown decreased tumor growth after IGF1R inactivation and with decreased circulating or tissue levels of IGF1 [121,148].

Studies of patients with acromegaly [149] or Laron dwarfisim [150] have been used as proxies to identify cancer risk in relation to IGF-1 excess or deficiency. They provide circumstantial evidence that increased IGF-1 levels are associated with cancer. Other forms of circumstantial evidence include the observation that height and birth weight, which is related to the concentration of IGF-1 in the umbilical cord, are related to the risk of some cancers [151-153]. Mammographic breast density, which is a strong risk factor for breast cancer is related to the level of circulating IGF-1 genes [154,155] and to polymorphisms in IGF-related genes [154,156].

Population based studies have provided evidence that relate circulating ligand levels as well as polymorphic variation of relevant genes to cancer risk and prognosis. Prospective epidemiologic studies provide evidence of a relationship between circulating IGF-1 and the risk of developing prostate, breast, colorectal and other cancers [142,143,157-161]. Individuals at the high end of the normal range of serum IGF-1 have more than double the risk of a subsequent cancer. Variability between studies, particularly regarding the potential reciprocal relationship between IGFBP3 and IGF-1 levels may be accounted for by technical challenges in measuring IGF and uncharacterised factors that modify IGF-1 levels including age and diurnal variation. Studies of single nucleotide polymorphisms in the IGF-1 axis genes indicate a potential relationship between SNPs and circulating levels [154,162] but increased risk of cancer development with IGF SNPs is unclear[163]. Studies report polymorphic variants associated with colorectal [164], oesophageal [165], ovarian [166] and non-small cell lung cancer [167] but not with breast or prostate cancer [168]. These studies require confirmation in other populations.

### IGF axis and obesity

Alterations in the IGF axis have been implicated in malignancies that are also associated with obesity, suggesting the IGF axis may play a mechanistic role in linking obesity and cancer. There is a paucity of studies examining the influence of obesity on IGF-1R in tumor tissue. A study in breast cancer demonstrated that increasing BMI was positively associated with increased IGF-1R expression in both normal mammary gland tissue and breast cancer tissue [169]. Furthermore research has found that IGF-1R expression is significantly higher in the colorectal neoplasms of individuals with metabolic syndrome than in the lesions of individuals without the syndrome [170]. The findings of these studies suggest that the molecular consequence of obesity is the increased expression of IGF-1R in both normal and malignant tissue.

As mentioned previously, elevated levels of free IGF-1 have been reported in obese individuals and are believed to be a consequence of hyperinsulinaemia inhibiting production of IGFBP-1 and -2[123,171]. The chronic systemic inflammation associated with obesity is believed to fuel tumor development and progression, and IGF-1 may mediate obesity-associated inflammation via its effects on immune cells, including macrophages. Studies have shown IGF-1 can lead to macrophage migration and invasion, and also increased macrophage production of proinflammatory cytokines [172,173]. In addition, recent research has supported a functional role for IGF-1 in obesity associated inflammation and tumorigenesis. In a murine model of obesity and chronic IGF-1 gene deficiency, diet-induced obese mice demonstrated increased local tumor growth and metastases compared to lean controls, but this was not seen in IGF-1 gene deficient mice. In addition, the expression of inflammatory cytokines and cell adhesion molecules was upregulated in obese compared to lean mice, but chronic IGF-1 deficiency was associated with a reduction in these indicators [174]. Hence it is possible that in obesity IGF-1 can affect tumor development both directly, by stimulating tumor growth and indirectly, by creating a microenvironment that is permissive for tumor growth.

## Summary and future directions

Obesity is likely to a causal factor for a number of common cancers, however, the mechanisms underpinning this association are not fully understood. Current hypotheses include the development of chronic inflammation with increased adiposity, which alters immune, sex steroid and adipokine function. In turn, alterations in insulin sensitivity and the IGF axis occur. Any or all of these systems act in concert to promote the development and progression of cancer at a cellular level. How these systemic conditions interact at the tumor site and whether their influence is site-specific, is a complex question. There has been little investigation of paracrine models of adipose tissue function in the obese state.

Some of the systemic changes associated with obesity are enhanced in the group of patients with visceral adiposity. Similar to the increased risk associated with central obesity and development of cardiovascular and metabolic disease, visceral fat may be more relevant to cancer development. Studies which measure central obesity as well as BMI may clarify whether total fat or visceral fat distribution is most relevant.

Future research using accurate definitions of obesity status are important in order to accurately determine the risk associated with obesity and whether the risk is specific to visceral fat.

## Competing interests

The authors declare that they have no competing interests.

## Authors' contributions

CLD and SLD drafted the manuscript, JVR conceived of the review and revised the manuscript. All authors have read and approved the final manuscript.
